# Prognostic role of high MTAP expression is reversed by the ERG status in prostate cancer treated by radical prostatectomy

**DOI:** 10.1016/j.neo.2025.101197

**Published:** 2025-06-18

**Authors:** Natalia Gorbokon, Seyma Büyücek, Henning Plage, Neele Heckmann, Ronald Simon, Maximilian Lennartz, Martina Kluth, Katharina Teljuk, Claudia Hube-Magg, Sarah Minner, Eike Burandt, Till S. Clauditz, Waldemar Wilczak, Guido Sauter, David Dum, Andrea Hinsch, Hans Heinzer, Alexander Haese, Thorsten Schlomm, Andreas M Luebke, Markus Graefen, Stefan Steurer, Christian Bernreuther, Luca Roma, Lukas Bubendorf, Sarah Weinberger

**Affiliations:** a Institute of Pathology, University Medical Center Hamburg-Eppendorf, Hamburg, Germany; b Department of Urology, Charité-Universitätsmedizin Berlin, Berlin, Germany; c Martini-Clinic, Prostate Cancer Center, University Medical Center Hamburg-Eppendorf, Hamburg, Germany; d Institute of Medical Genetics and Pathology, University Hospital Basel, Basel, Switzerland

**Keywords:** MTAP, Prostate cancer, Tissue microarray, Prognosis, TMPRSS2:ERG fusion, Immunohistochemistry

## Abstract

Loss of S-methyl-5′-thioadenosine phosphorylase (MTAP) expression offers a therapeutic option through synthetic lethality and confers resistance to immune checkpoint inhibitors in various cancers. To assess MTAP prevalence in prostate cancer, a tissue microarray of 17,747 samples was analyzed via immunohistochemistry. Normal prostate glands showed weak to moderate cytoplasmic MTAP staining. In 13,189 interpretable cancers, a complete loss of MTAP staining was seen in 33 (0.3 %) tumors, while MTAP staining was considered 1+ in 14.8 %, 2+ in 42.2 %, and 3+ in 42.7 % of tumors. Fluorescence in situ hybridization analysis of 9 MTAP-negative cancers confirmed homozygous MTAP deletion in all of these tumors. MTAP staining was significantly stronger in cancers harboring the *TMPRSS2:ERG* fusion than in ERG fusion negative tumors (*p* < 0.0001). A comparison with clinico-pathological features revealed inverse correlations depending on the ERG fusion status: In ERG-negative cancers, high (3+) MTAP expression correlated with advanced pT stage, high Gleason grade, and early PSA recurrence (*p* < 0.0001 each). Conversely, in ERG-positive tumors, MTAP expression decreased with advanced pT stage (*p* < 0.0001), high classical (*p* = 0.0004) and quantitative Gleason grade (*p* = 0.0005), and low (1+) MTAP expression was significantly linked to early PSA recurrence (*p* = 0.0012). Comparison with 11 previously analyzed chromosomal deletions identified ERG-status-dependent positive or negative associations between MTAP expression and deletions of PTEN and 12p13 (*p* ≤ 0.0274), suggesting functional interactions. Taken together, the results of our study demonstrate that MTAP deficiency is exceedingly rare in prostate cancer, while high MTAP expression is a strong and independent marker for poor prognosis in ERG negative cancers.

## Introduction

The enzyme S-methyl-5′-thioadenosine phosphorylase (MTAP) is of topical interest because its inactivation by homozygous deletion occurs commonly in cancer and offers several new therapeutic options. MTAP is critical to produce adenine, a critical component of both DNA and RNA and therefore needed for cell replication. Adenine can only be built by two mechanisms: either by MTAP-independent de novo synthesis or by recycling from adenine containing molecules through the MTAP-dependent salvage pathway [1]. In most cancer types, MTAP deficiency is caused by homozygous co-deletion with cyclin dependent kinase inhibitor 2A (CDKN2A) gene at 9p21.3 which is homozygously deleted in up to 15 % of all human cancers [[2], [3], [4]]. In MTAP deficient tumor cells, adenine synthesis is solely dependent on de novo biosynthesis which directly or indirectly involves several druggable enzymes including dihydrofolate reductase and phosphoribosylglycinamide formyltransferase [5]. MTAP deficient cells can also be targeted by drugs inhibiting protein arginine N-methyltransferase 5 (PRMT5) and methionine adenosyltransferase II, alpha **(**MAT2A) (summarized in [6]). PRMT5 is an essential enzyme for methylation of numerous proteins [7] whilst MAT2A is essential for the synthesis of S-adenosylmethionine (SAM), the methyl donor and substrate of PRMT5 [8,9]. PRMT5 is upregulated and involved in the progression of various cancers (summarized in [6]). Because PRMT5 is partially inhibited by the intracellular accumulation of the unprocessed MTAP metabolite 5′-deoxy-5′-methylthioadenosine (MTA) [10], MTAP deficiency makes cells vulnerable to drugs targeting PRMT5 or MAT2A (summarized in [6]). A clinical phase 1 trial has shown significant size reduction of MTAP-deleted cancers after treatment with the PRMT5 inhibitor MRTX1719 [11]. Other clinical trials employing drugs targeting PRMT5 are ongoing (summarized in [12]).

Although homozygous 9p21 deletions have been reported to occur in up to 40 % of prostate cancers [[13], [14], [15], [16], [17], [18], [19]], most more recent studies suggested markedly lower rates. A query of the cBioportal database (05.06.2024) [17] including 2,565 prostate cancers with copy number profiling from 24 studies revealed 33 (1.24 %) tumors with “deep” (largely corresponding to a homozygous deletion) MTAP deletion. Using fluorescence in situ hybridization (FISH), the gold standard method for detection of copy number aberrations in cancer, de Barros et al. [16] found homozygous 9p21 deletions in 8 of 111 prostate cancers and described an unfavorable disease course in these patients. However, Bistulfi et al. [15] did not find evidence for MTAP expression loss in 13 cell lines and 75 clinical samples of prostate cancer but reported evidence for an important functional role of MTAP in prostate cancer cells including a blockade of androgen sensitive prostate cancer growth by MTAP inhibition in vitro and in vivo. In a follow-up study, the same group further reported increased apoptosis after ex vivo treatment of human prostate cancers by a combinatorial treatment including the MTAP inhibitor Methylthio-DADMe-Immucillin-A (MTDIA).

Considering the discrepant data on the prevalence of MTAP deficiency and evidence for a clinical importance of MTAP expression in prostate cancer, a preexisting tissue microarray (TMA) containing more than 17,000 prostate cancer specimens with annotated clinical data was analyzed by immunohistochemistry (IHC) for MTAP expression using a previously validated assay [20].

## Materials and Methods

### Patients

Radical prostatectomy specimens were available from 17,747 patients, undergoing surgery between 1992 and 2015 at the Department of Urology and the Martini Clinic at the University Medical Center Hamburg-Eppendorf. All prostate specimens were analyzed according to a standard procedure, including a complete embedding of the entire prostate for histological analysis [21]. Available histopathological data included tumor stage, Gleason grade, nodal stage and resection margin status. In addition to the classical Gleason categories, “quantitative” Gleason grading was performed as described before [22]. In brief, for every prostatectomy specimen, the percentages of Gleason 3, 4, and 5 patterns were recorded in cancerous tissues in addition to the regular process of Gleason grading. Gleason 3 + 4 and 4 + 3 cancers were divided into subgroups according to their percentage of Gleason 4 pattern. For practical use, we subdivided the 3 + 4 and 4 + 3 cancers in 8 subgroups: 3 + 4 ≤ 5 % Gleason 4, 3 + 4 6-10 %, 3 + 4 11-20 %, 3 + 4 21-30 %, 3 + 4 31-49 %, 4 + 3 50-60 %, 4 + 3 61-80 % and 4 + 3 > 80 % Gleason 4. Two additional groups were defined by the presence of a tertiary Gleason 5 pattern, including 3 + 4 Tert.5 and 4 + 3 Tert.5. Follow-up data were available for 14,464 patients with a median follow-up of 48 months (Supplementary Table 1). PSA values were regularly measured after surgery and PSA recurrence was defined as the time point when postoperative PSA was at least 0.2 ng/ml and increasing at subsequent measurements. The TMA manufacturing process was described as before [23]. One 0.6 mm core was taken from a tumor containing tissue block from each patient. The attached molecular database contained data on Ki67 Labeling Index (Ki67LI) of 5,492 tumors [24], ERG expression data of 13,089 tumors [25], *ERG* break apart FISH data of 7,225 (expanded from [25]), androgen receptor (AR) expression data of 7,971 cancers [26] as well as data on the deletion status of 5q21 (*CHD1*) of 8,047 (expanded from [27]), 6q15 (*MAP3K7*) of 6,171 (expanded from [28]), *PTEN* (10q23) of 6,803 (expanded from [29]), 3p13 (*FOXP1*) of 7,201 (expanded from [30]), 13q14 of 7,499 [31], 18q21 of 7,032 [32], 8p21 of 7,001 [33], 12p13 of 6,187 [34], 12q24 of 7,435 [25], 16q24 of 5,493 [35], and 17p13 (*TP53*) of 8,307 cancers [36]. The use of archived diagnostic left-over tissues for manufacturing of tissue microarrays and their analysis for research purposes as well as patient data analysis has been approved by local laws (HmbKHG, §12,1) and by the local ethics committee (Ethics commission Hamburg, WF-049/09 and PV3652). All work has been carried out in compliance with the Helsinki Declaration.

### Immunohistochemistry (IHC)

A TMA containing bladder carcinomas with 9p21 wild type (*n* = 20), heterozygous deletions (*n* = 20) and homozygous deletion (*n* = 20) was used to titrate the antibodies to obtain maximal staining in non-neoplastic cells while background staining was still lacking in homozygously deleted cancers. The optimal protocol was as follows. Freshly prepared 2.5 µm TMA sections were applied on one day in one experiment in a Dako Omnis automated stainer (Agilent Technologies, Santa Clara, CA, USA) using the EnVision FLEX, High pH Kit (Agilent Technologies, Santa Clara, CA, USA, #GV800). Slides were deparaffinized with Clearify^TM^ agent (Agilent Technologies, Santa Clara, CA, USA, #GC810) and exposed to heat-induced antigen retrieval for 30 minutes at 97°C in Target Retrieval Solution, High pH reagent (part of Agilent kit #GV800). Primary antibody specific for MTAP (recombinant rabbit monoclonal, MSVA-741R, MS Validated Antibodies GmbH, Hamburg, Germany, #5293-741R) was applied at ambient temperature for 30 minutes at a dilution of 1:50. Endogenous peroxidase activity was blocked with Peroxidase-Blocking-Reagent (part of Agilent kit #GV800) for 3 minutes. Bound antibody was visualized using the EnVision FLEX, High ph kit reagents DAB+ Chromogen and Substrate Buffer (parts of Agilent kit #GV800) and EnVision FLEX + Rabbit linker (Agilent Technologies, Santa Clara, CA, USA; #GV809) according to the manufacturer’s directions. The sections were counterstained with hemalaun. For tumor tissues, the average staining intensity of unequivocally neoplastic cells was estimated as 0, 1+, 2+, 3+. For the classification of a tumor as completely negative (0), presence of unequivocal MTAP staining in tumor adjacent normal tissue was required. Tumors with complete absence of MTAP staining in cancerous cells and a lack of stromal cells with unequivocal MTAP staining were considered “non-informative”. Examples of prostate cancers with different staining intensities are shown in Supplementary Figure 1.

### Fluorescence in-situ hybridization (FISH)

FISH was executed as previously described [37]. In brief, five micrometer TMA sections were deparaffinized, rehydrated and exposed to heat-induced denaturation for 10 minutes in a water bath at 99°C in P1 pretreatment solution (Agilent Technologies, Santa Clara, CA, USA; #K5799). For proteolytic treatment, slides were added to VP2000 protease buffer (Abbott, Chicago, IL, USA; #2 J.0730) for 200 minutes at 37°C in a water bath. A commercial FISH probe kit containing both, a 9p21 probe including both the *CDKN2A* and the *MTAP* gene and a centromere 9 probe were utilized for 9p21 copy number detection (ZytoLight ® SPEC CDKN2A/CEN 9 Dual Color Probe, Zytovision, Bremerhaven, Germany; #Z-2063). Hybridization was performed overnight at 37°C. Posthybridization washes were done according to the manufacturer’s direction at 37°C. Stained tissues were manually interpreted and copy numbers of 9p21 and centromere 9 were estimated for each tissue spot. Presence of roughly equal numbers of 9p21 and centromere 9 signals in tumor cell nuclei was considered as 9p21 normal. Presence of fewer 9p21 signals than centromere 9 probe signals in at least 60 % tumor cell nuclei or one 9p21 and one centromere 9 signal (monosomy of chromosome 9) in nearly all tumor cell nuclei were considered as heterozygous deletion. Complete absence of 9p21 signals but presence of centromere 9 signals in the tumor cell nuclei and of unequivocal 9p21 signals in tumor adjacent normal cell nuclei, was considered a homozygous 9p21 deletion. Tissue spots lacking 9p21 signals in all (tumor and normal cell) nuclei were excluded from analysis.

### Statistics

Statistical calculations were performed with JMP17 software (SAS Institute Inc., NC, USA). Contingency tables and the chi²-test were performed to search for associations between molecular parameters and tumor phenotype. Survival curves were calculated according to Kaplan-Meier. The Log-Rank test was applied to detect significant differences between groups. Cox proportional hazards regression analysis was performed to test the statistical independence and significance between pathological, molecular and clinical variables. Separate analyses were performed using different sets of parameters available either before or after prostatectomy.

## Results

### Technical issues

A total of 13,189 (74.3 %) tumor samples were interpretable in our TMA analysis. Non-informative cases (*n* = 4,558; 25.7 %) lacked MTAP staining on both tumor and stromal cells (IHC), unequivocal tumor cells on the TMA spots, or entire tissue spots on the TMAs.

### MTAP expression in normal and cancerous prostate tissues

**Figure 1:**
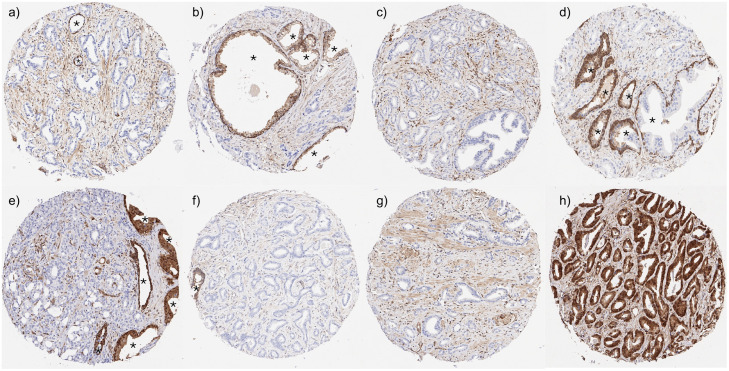
Representative images of MTAP immunostainings in normal and cancerous prostate tissues. The panels a-g show cancers with MTAP expression loss in the tumor cells but retained MTAP expression in the surrounding stroma and normal prostate glands (marked by an *). Panel h shows a prostate cancer with retained MTAP expression in the tumor cells.

Normal prostate glands showed a weak to moderate, predominantly cytoplasmic MTAP positivity of epithelial cells. In cancers, a complete loss of MTAP expression (MTAP deficiency) was seen in 33 (0.3 %) of informative samples, while the MTAP staining was considered 1+ in 14.8 %, 2+ in 42.2 %, and 3+ in 42.7 % of tumors. Samples with adjacent normal and cancerous glands showed that both an increased and a decreased MTAP expression were possible in cancer cells as compared to normal prostate glands. Representative images of MTAP immunostainings in normal and cancerous glands are shown in Figure 1. Additional cases with complete MTAP expression loss are shown in Supplementary Figure 2.

### MTAP FISH validation

FISH validation of 9 MTAP deficient cases revealed a homozygous 9p21 deletion in all 9 cases (Supplementary Figure 3).

### *TMPRSS2:ERG* fusion status and ERG protein expression

**Figure 2:**
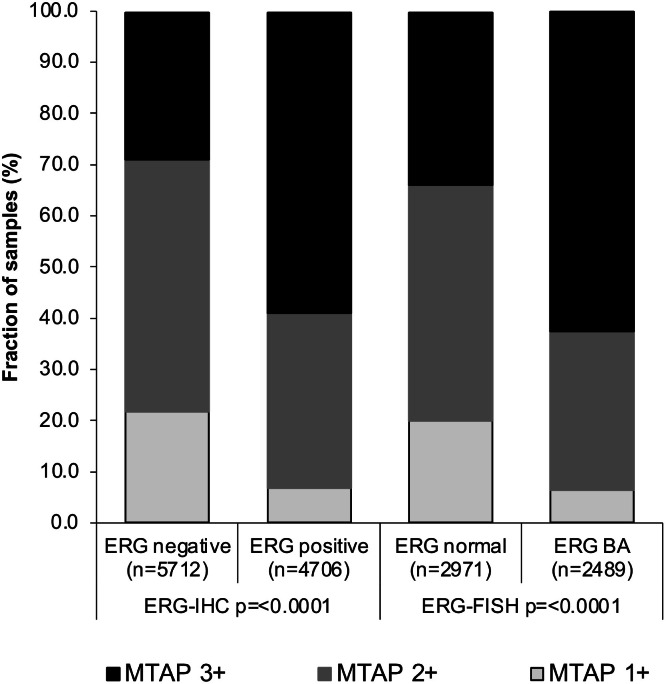
MTAP expression and ERG fusion status by IHC and FISH.

Of the tumors with interpretable MTAP staining, 5,460 had data on ERG FISH and 10,418 cancers had ERG IHC data available. A higher level of MTAP staining was linked to *TMPRSS2:ERG* fusion positive prostate cancers (*p* < 0.0001; Figure 2). For example, 2+ to 3+ MTAP staining was seen in 78 % of ERG IHC negative, but in 92.8 % of ERG IHC positive cancers. There was no significant difference in the ERG status between the 29 MTAP deficient (44.8 % ERG positive by IHC) and the 10,389 non-MTAP deficient cancers (45.2 % ERG positive, *p* = 0.9702).

### Tumor phenotype and PSA recurrence

**Figure 3:**
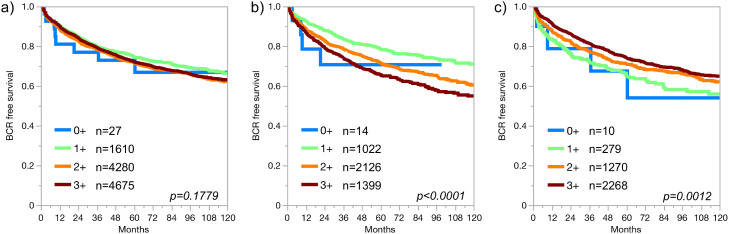
MTAP expression and prostate cancer prognosis in a) all cancers, b) ERG-negative cancers and c) ERG-positive cancers.

MTAP expression was significantly linked to pT stage (*p* = 0.0004, Supplementary Table 2), classical and quantitative Gleason grade (*p* < 0.0001 each), and presence of lymph node metastasis (*p* = 0.0315, but was unrelated to PSA recurrence (*p* > 0.1779, Figure 3a). Considering the strong difference in MTAP expression between ERG positive and ERG negative cancers, these subgroups were separately analyzed for the role of MTAP expression. Strikingly, the relationship between MTAP expression and aggressive tumor phenotype became much clearer in these subgroups although inverse relations were observed. In ERG negative carcinomas, the levels of MTAP expression increased markedly with advanced pT stage and classical or quantitative Gleason grade (*p* < 0.0001 each, Table 1), and high (3+) MTAP expression was strongly linked to early PSA recurrence (*p* < 0.0001, Figure 3b). In ERG positive cancers, MTAP expression decreased with advanced pT stage (*p* < 0.0001), classical (*p* = 0.0004) and quantitative Gleason grade (*p* = 0.0005, Table 2), and low MTAP expression was significantly linked to early PSA recurrence (*p* = 0.0012, Figure 3c).

**Table 1:** Associations between MTAP expression and prostate cancer phenotype in ERG negative cancers.

			MTAP IHC result				
		n evaluable	0+	1+	2+	3+	p value
**all ERG negative cancers**		5,712	0.3	21.7	49.4	28.6	
**Tumor stage**	pT2	3,734	0.2	23.6	49.3	26.9	<0.0001
	pT3a	1,172	0.5	19.4	49.4	30.7	
	pT3b-4	781	0.3	16.3	49.7	33.8	
**Gleason grade**	≤3 + 3	945	0.1	33.1	41.3	25.5	<0.0001
	3 + 4	2,996	0.4	21.6	50.1	27.9	
	3 + 4 Tert.5	292	0	17.5	54.8	27.7	
	4 + 3	628	0.2	17.4	51.4	31.1	
	4 + 3 Tert.5	421	0.2	13.5	51.5	34.7	
	≥4 + 4	380	0.3	14.5	52.1	33.2	
**quantitative Gleason**	3 + 4 ≤ 5 %	732	0.4	22	50.3	27.3	<0.0001
	3 + 4 6-10 %	746	0.5	22.7	46.8	30	
	3 + 4 11-20 %	655	0.5	18.8	51.9	28.9	
	3 + 4 21-30 %	348	0	19.5	53.7	26.7	
	3 + 4 31-49 %	295	0.7	22.4	47.5	29.5	
	4 + 3 50-60 %	243	0	17.5	54.8	27.7	
	4 + 3 61-80 %	224	0.4	17.7	52.3	29.6	
	4 + 3 > 80 %	72	0	16.1	49.6	34.4	
**Lymph node metastasis**	N0	3,577	0.3	20.3	50.2	29.2	0.0968
	*N*+	412	0.2	15.3	52.9	31.6	
**Preop. PSA level (ng/ml)**	<4	569	0.4	19.5	42	38.1	<0.0001
	4-10	3,251	0.3	21.5	49.1	29.1	
	11-20	1,358	0.2	22.3	51.4	26.1	
	>20	503	0.2	23.7	53.5	22.7	
**Surgical margin**	negative	4,503	0.3	22.2	49.1	28.4	0.3297
	positive	1,188	0.3	19.8	50.3	29.6

**Table 2:** Associations between MTAP expression and prostate cancer phenotype in ERG positive cancers.

			MTAP IHC result				
		n evaluable	0+	1+	2+	3+	p value
**all ERG positive cancers**		4,706	0.3	6.9	34.2	58.6	
							
**Tumor stage**	pT2	2,634	0.1	6.2	31.9	61.7	<0.0001
	pT3a	1,299	0.4	6.4	36.1	57.1	
	pT3b-4	748	0.7	10	39	50.3	
**Gleason grade**	≤3 + 3	831	0.1	7.8	30.9	61.1	0.0004
	3 + 4	2,570	0.2	6.1	33	60.7	
	3 + 4 Tert.5	190	0.5	5.3	35.8	58.4	
	4 + 3	483	0.4	8.5	38.3	52.8	
	4 + 3 Tert.5	335	0	8.1	39.1	52.8	
	≥4 + 4	240	1.3	10	40	48.8	
**quantitative Gleason**	3 + 4 ≤ 5 %	595	0.2	5.9	29.2	64.7	0.0005
	3 + 4 6-10 %	634	0.2	5.7	30.4	63.7	
	3 + 4 11-20 %	565	0.2	5.8	32.7	61.2	
	3 + 4 21-30 %	300	0.3	5.3	41	53.3	
	3 + 4 31-49 %	241	0	9.5	34.9	55.6	
	4 + 3 50-60 %	203	0.5	5.3	35.8	58.4	
	4 + 3 61-80 %	164	0	6.4	36.5	57.1	
	4 + 3 > 80 %	31	0.6	11	34.1	54.3	
**Lymph node metastasis**	N0	2,769	0.2	6.4	35.4	58	0.0008
	*N*+	426	0.9	9.2	40.6	49.3	
**Preop. PSA level (ng/ml)**	<4	593	0.3	5.7	31.4	62.6	<0.0001
	4-10	2,817	0.3	6.3	32.8	60.6	
	11-20	931	0.3	8.3	38	53.4	
	>20	325	0	9.8	40.9	49.2	
**Surgical margin**	negative	3,601	0.3	6.9	33.5	59.3	0.291
	positive	1,081	0.3	6.8	36.6	56.2	

### Multivariate analysis

In both ERG negative and ERG positive cancers, four different multivariate analyses were performed to evaluate the clinical relevance of MTAP expression in different scenarios (Supplementary Table 3). Scenario 1 evaluated all postoperatively available parameters including pT, pN, surgical margin status, preoperative PSA value and Gleason grade obtained on the prostatectomy specimen. In scenario 2, all postoperatively available parameters except pN were included. The rational for this approach was that indication and extent of lymph node dissection is not standardized in the surgical therapy of prostate cancer and may introduce a bias towards inclusion of more high-grade cancers. Two additional scenarios were to model the preoperative situation as accurately as possible: Scenario 3 included MTAP expression, preoperative PSA, clinical tumor stage (cT stage) and Gleason grade obtained on the prostatectomy specimen. Since postoperative determination of a tumor’s Gleason grade is more precise than the preoperatively determined Gleason grade (subjected to sampling errors and consequently under-grading in more than one-third-of cases) this parameter was replaced by the preoperative Gleason grade obtained on the original biopsy in Scenario 4. MTAP expression provided significant prognostic value beyond the established parameters in all of the described scenarios in the subgroup of ERG negative cancers but not in ERG positive cancers.

### Androgen receptor, Ki67 labeling index (LI), and chromosomal deletions

Data on both MTAP and AR were available for 6,187 cancers. There was a strong positive association between AR expression and MTAP expression in all cancers as well as in subsets of ERG negative and ERG positive cancers (*p* < 0.0001 each; Supplementary Figure 4). High MTAP expression levels were strongly related to a high Ki67LI in 2,442 ERG negative but not in 2,229 ERG positive cancers. Among ERG negative cancers, Ki67LI was 1.7 ± 0.1 in 584 tumors with 1+, 2.7 ± 0.1 in 1,103 tumors with 2+, and 3.3 ± 0.1 in 747 tumors with 3+ MTAP staining (*p* < 0.0001). Significant associations were also retained in most subgroups with identical quantitative Gleason grade (Table 3). The relationship of MTAP expression with 11 of the most frequent genomic deletions (10q23/*PTEN*, 3p13, 5q21, 6q15, 13q14, 18q21, 8p21, 12p13, 12q24, 16q24, 17p13) is shown for the subgroups of ERG positive and negative cancers in Supplementary Figure 5. In ERG negative cancers, high MTAP expression was related to deletions of *PTEN* (*p* < 0.0001), 8p21 (*p* < 0.0001), 5q21 (*p* = 0.0049), 12p13 (*p* = 0.0274), and 17p13 (*p* = 0.001). In ERG positive cancers, low MTAP expression was associated with deletions of *PTEN* (*p* = 0.0002), 12p13 (*p* < 0.0001), and 18q21 (*p* = 0.0006).

**Table 3:** Associations between MTAP expression and tumor cell proliferation measured by KI67 immunohistochemistry in ERG negative and ERG positive cancers.

	**MTAP**	**ERG negative cancers**			**ERG positive cancers**		
		n	Ki67 Av±SD	P	n	Ki67 Av±SD	P
**All Gleason**	**0+**	8	2.6 ± 0.9		5	3.2 ± 1.2	
**grades**	**1+**	584	1.7 ± 0.1	<0.0001	172	2.6 ± 0.2	0.4695
	**2+**	1,103	2.7 ± 0.1		695	2.8 ± 0.1	
	**3+**	747	3.3 ± 0.1		1357	2.9 ± 0.1	
**≤3+3**	**0+**	0	0 ± 0		2	1 ± 1.4	
	**1+**	189	1.4 ± 0.1	<0.0001	37	1.9 ± 0.3	0.4504
	**2+**	177	1.9 ± 0.1		141	2.4 ± 0.2	
	**3+**	108	2.6 ± 0.2		311	2.4 ± 0.1	
**3+4**	**0+**	6	2.8 ± 0.9		2	5 ± 1.7	
	**1+**	288	1.6 ± 0.1	<0.0001	94	2.5 ± 0.2	0.09
	**2+**	591	2.3 ± 0.1		418	2.7 ± 0.1	
	**3+**	417	2.9 ± 0.1		799	2.9 ± 0.1	
**3+4 tert5**	**0+**	0	0 ± 0		0	0 ± 0	
	**1+**	13	1.1 ± 0.7	0.0041	4	1.8 ± 1.3	0.3908
	**2+**	73	3.6 ± 0.3		15	3 ± 0.7	
	**3+**	37	3.5 ± 0.4		47	3.5 ± 0.4	
**4+3**	**0+**	1	2 ± 3.6		2	2.5 ± 2	
	**1+**	47	2.5 ± 0.5	0.2711	22	3.5 ± 0.6	0.9641
	**2+**	142	3.4 ± 0.3		66	3.2 ± 0.3	
	**3+**	84	3.8 ± 0.4		116	3.3 ± 0.3	
**4+3 tert5**	**0+**	0	0 ± 0		0	0 ± 0	
	**1+**	21	1.1 ± 0.9	0.0008	8	4.3 ± 1.2	0.3788
	**2+**	59	3.7 ± 0.5		28	4.2 ± 0.7	
	**3+**	53	5 ± 0.5		52	3.1 ± 0.5	
**≥4+4**	**0+**	1	2 ± 4		0	0 ± 0	
	**1+**	26	3.7 ± 0.8	0.6065	7	4 ± 2.5	0.851
	**2+**	60	4.3 ± 0.5		26	4.4 ± 1.3	
	**3+**	46	4.8 ± 0.6		32	5.2 ± 1.2	

## Discussion

The analysis of 13,189 prostate cancers identified only 33 cases (0.3 %) with a complete MTAP loss, indicating that MTAP deficiency is a rare event in prostate cancer. These data are in line with two earlier IHC studies finding a complete MTAP expression loss in 0 of 75 [15] and in 0.8 % of 361 prostate cancers [20]. FISH validation not only confirmed the MTAP deficiency in all 9 selected cases but also demonstrated that the MTAP expression loss in prostate cancer is indeed caused by homozygous 9p21 deletions. In a study on 13,067 cancers from 149 different tumor types we had earlier found that MTAP deficiency is unrelated to 9p21 deletions in several tumor types including malignant lymphoma and neuroendocrine neoplasms of various organs [20]. In the same study, our MTAP IHC assay had been extensively validated according to the guidelines of the international working group for antibody validation [38] and resulted in a 90-100 % concordance with homozygous deletion depending on the tumor type [20]. The absence of associations with tumor phenotype and PSA recurrence seen in this study argues against a major clinical impact of MTAP deficiency in prostate cancer.

The significant association of increased MTAP expression levels with increased tumor cell proliferation, high grade, advanced pT stage and early PSA recurrence in cancers lacking a *TMPRSS2:ERG* fusion is in line with the known function of MTAP as a critical enzyme for adenine synthesis which is required for DNA and RNA synthesis in dividing cells. Our findings - including the significant association with AR expression - are also in agreement with functional evidence for androgenic regulation of MTAP expression [39,40] and a role of MTAP for prostate cancer cell growth [15]. The prognostic role of MTAP being independent of all preoperatively and postoperatively available histopathological prognostic features may even point towards a potential clinical application of MTAP measurement in ERG negative cancer.

The reversal of all these expectable MTAP associations with tumor phenotype and patient outcome in cancers carrying an *TMPRSS2:ERG* fusion is the most striking finding of this study.

The *TMPRSS2:ERG* fusion represents the most common genomic alteration in prostate cancer, occurring in almost half of all prostate cancers especially in younger patients [41]. The fusion of the regulatory elements of the androgen regulated TMPRSS2 gene with the ERG gene results in a permanent androgen dependent overexpression of the transcription factor ERG. ERG expression lacks prognostic relevance [42] but modifies the expression of more than 1,600 genes in prostate epithelial cells [43]. It is therefore not uncommon that the prognostic impact of molecular factors varies between ERG positive, and ERG negative cancers. For example, loss of CEACAM1 [44], SOX9 [45], phospho-mTOR [46], and CEBPA [47], as well as deletion at chromosome 3p13 [30] (displayed a prognostic role only in ERG-positive cancers, while altered expression of SFRP4 [48], GATA2 [49], hnRNPA1 [50], NBS1 [51], FOXP2 [52], MTC02 [53], BCAR1 [54], p16 [55], YB-1 [56], MSH6 [57], and GGH [58] were prognostically relevant in ERG-negative tumors only.

The mechanism by which ERG expression reversed the role of MTAP in prostate cancer cells is unclear and cannot be determined from our data. However, it is tempting to speculate that this effect could result from the known interaction between ERG and PRMT5 that can only occur in *TMPRSS2:ERG* fusion positive prostate cancers. PRMT5 activity depends on functional MTAP [6] and processes the large amounts of methionine that originate from the MTAP-dependent methionine salvage cycle [15]. It was shown that ERG recruits PRMT5 to AR target genes, where PRMT5 attenuates AR-dependent transcription by methylating AR at arginine 761 [59]. In the presence of an *TMPRSS2:ERG* fusion, it is therefore conceivable that tumors with low MTAP expression may be better able to maintain the AR-dependent growth processes than tumors with high MTAP levels.

Our findings do also allow for therapeutic speculations. The virtual independency of the adenine requiring proliferative activity of ERG positive tumor cells from MTAP expression levels might suggest that the MTAP dependent salvage pathway for adenine synthesis has reduced importance in these cancers. If this holds true, the use of PRMT5 or MAT2A inhibitors, as suggested by Bistulfi et al. [15], might be substantially less efficient in ERG positive than in ERG negative cancers. Assuming further that ERG positive cancers might in turn be more dependent on de novo adenine biosynthesis, it would be interesting to learn whether targeting druggable enzymes of this pathway might be efficient in ERG positive prostate cancer.

Chromosomal deletions represent the next most common recurrent genomic alterations in prostate cancer after *TMPRSS2:ERG* fusions. Except from for *PTEN* on 10q23 [60] or *NKX3.1* on 8p21 [33,61], critical target genes of these deletions are not known. It is assumed that a reduced expression of multiple genes jointly exerts a tumor promoting effect in deleted cancer cells through haploinsufficiency [14,62]. Because most deletions are markedly more frequent in either ERG positive (such as 3p14, 8p21, *PTEN*, 12p13, 16q24, 17p13) or ERG negative (5q13, 5q21, 6q15, 13q14) cancers [25,[27], [28], [29], [30], [31], [32], [33], [34], [35], [36]] and MTAP was also associated with the ERG status, the relationship between MTAP and specific deletions was separately analyzed in ERG positive and ERG negative cancers. That both *PTEN* and 12p13 deletions showed the same inverse relationship with MTAP expression as seen for tumor cell proliferation raises the possibility that *PTEN* and one or several genes on 12p13 (such as the p27/KIP1 tumor suppressor) may be directly or indirectly involved in the ERG dependent switch of the MTAP role in prostate cancer. Functional interactions of *PTEN* deletions and MTAP or its interdependent enzymes in the methionine cycle or the methionine salvage pathway are not known, however, the level of the MTAP substrate methylthioadenosine (MTA) has been shown to impact cell proliferation by regulating p27 expression in normal liver epithelial cells [63].

In summary, the IHC analysis of 13,189 prostate cancers revealed that MTAP deficiency is exceedingly rare, that high MTAP expression is a strong and statistically independent marker for poor prognosis in ERG negative cancers, and that ERG activation fully reverses these MTAP effects even linking high MTAP expression to favorable outcome. All these findings are in line with a significant functional role of MTAP in prostate cancer cells which deserves further investigation.

## CRediT authorship contribution statement

**Natalia Gorbokon:** Conceptualization, Data curation, Formal analysis, Project administration, Supervision, Writing – original draft. **Seyma Büyücek:** Data curation, Visualization, Writing – review & editing. **Henning Plage:** Formal analysis, Resources, Writing – review & editing. **Neele Heckmann:** Data curation, Resources, Writing – review & editing. **Ronald Simon:** Conceptualization, Formal analysis, Project administration, Supervision, Writing – original draft. **Maximilian Lennartz:** Formal analysis, Resources, Validation, Writing – review & editing. **Martina Kluth:** Conceptualization, Data curation, Formal analysis, Project administration, Supervision, Writing – original draft. **Katharina Teljuk:** Data curation, Formal analysis, Resources, Writing – review & editing. **Claudia Hube-Magg:** Formal analysis, Visualization, Writing – review & editing. **Sarah Minner:** Resources, Writing – review & editing. **Eike Burandt:** Data curation, Writing – review & editing. **Till S. Clauditz:** Data curation, Resources, Writing – review & editing. **Waldemar Wilczak:** Data curation, Resources, Writing – review & editing. **Guido Sauter:** Conceptualization, Investigation, Project administration, Supervision, Writing – original draft. **David Dum:** Data curation, Resources, Writing – review & editing. **Andrea Hinsch:** Data curation, Formal analysis, Writing – review & editing. **Hans Heinzer:** Resources, Writing – review & editing. **Alexander Haese:** Resources, Writing – review & editing. **Thorsten Schlomm:** Data curation, Resources, Writing – review & editing. **Andreas M Luebke:** Resources, Writing – review & editing. **Markus Graefen:** Data curation, Resources, Writing – review & editing. **Stefan Steurer:** Data curation, Resources, Writing – review & editing. **Christian Bernreuther:** Data curation, Resources, Validation, Writing – review & editing. **Luca Roma:** Data curation, Resources, Writing – review & editing. **Lukas Bubendorf:** Data curation, Resources, Writing – review & editing. **Sarah Weinberger:** Conceptualization, Project administration, Supervision, Writing – original draft.

## Declaration of competing interest

The authors declare the following financial interests/personal relationships which may be considered as potential competing interests: The recombinant rabbit monoclonal antibody, clone MSVA-741R was provided from MS Validated Antibodies GmbH, Hamburg, Germany (owned by a family member of GS)

## Appendix Supplementary materials

**Supplementary Figure 1**: Examples of prostate cancers with A) negative (intensity 0), B) 1+, C) 2+ and D) 3+ staining intensity. Image, application 1

**Supplementary Figure 2**: Further examples of prostate cancers with complete MTAP expression loss. Image, application 2

**Supplementary Figure 3**: Examples of 9p21 FISH analysis in prostate cancer. a) normal 9p21 copy numbers as indicated by the presence of two green 9p21 locus specific probe signals and two orange centromer 9 probe signals. b) Homozygous 9p21 loss as indicated by the complete absence of the green 9p21 locus specific probe signals. Cell nuclei were couterstained with DAPI (blue color). Image, application 3

**Supplementary Figure 4:** MTAP expression and AR expression. Image, application 4

**Supplementary Figure 5:** MTAP expression and genomic deletions in a) ERG negative and b) ERG positive cancers. Image, application 5

**Supplementary Table 1.** Study cohort Image, application 6

**Supplementary Table 2:** Associations between MTAP expression and prostate cancer phenotype in all cancers. Image, application 7

**Supplementary Table 3:** Multivariate analysis. Cox proportional hazards regression p-values are shown for established prognostic parameters and MTAP expression in different clinical scenarios and in subsets of ERG-negative and ERG-positive cancers. Image, application 8
