# Supplementary figures and images for: Prognostic role of high MTAP expression is reversed by the ERG status in prostate cancer treated by radical prostatectomy
Source: Neoplasia. 2025 Jun 18;67:101197. doi: 10.1016/j.neo.2025.101197 (PMC12214127; doi:10.1016/j.neo.2025.101197)

A

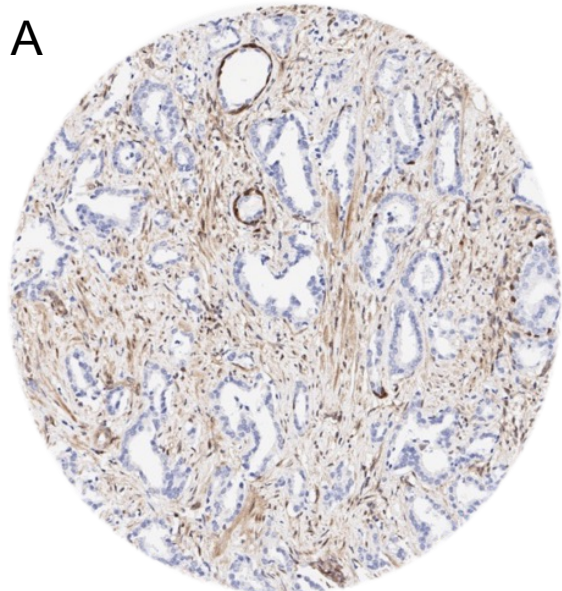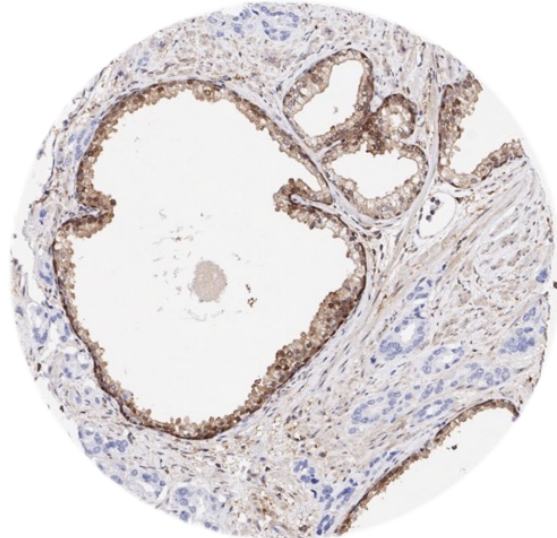

B

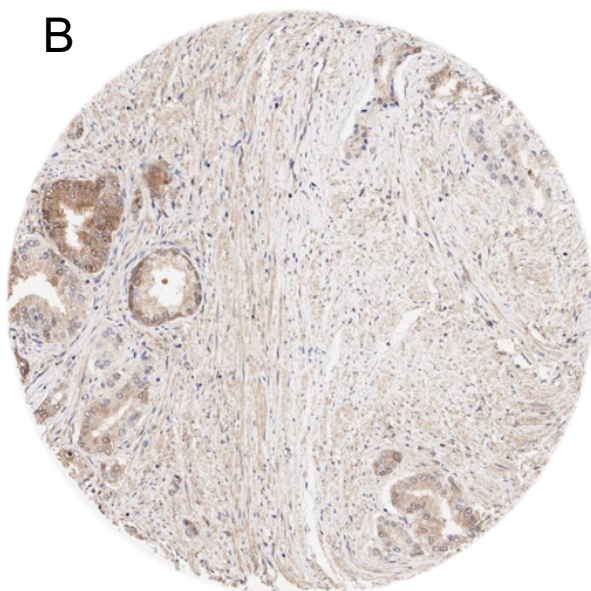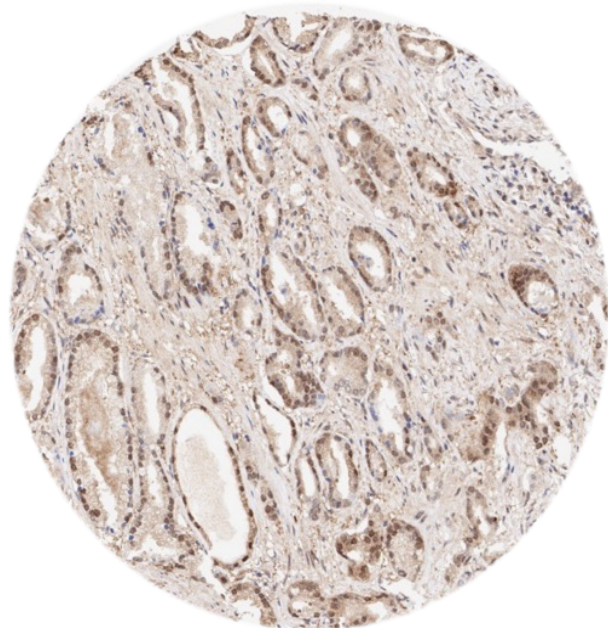

C

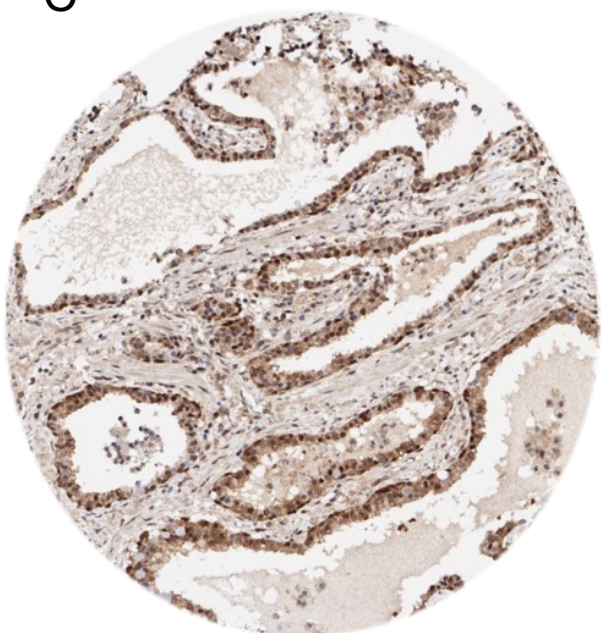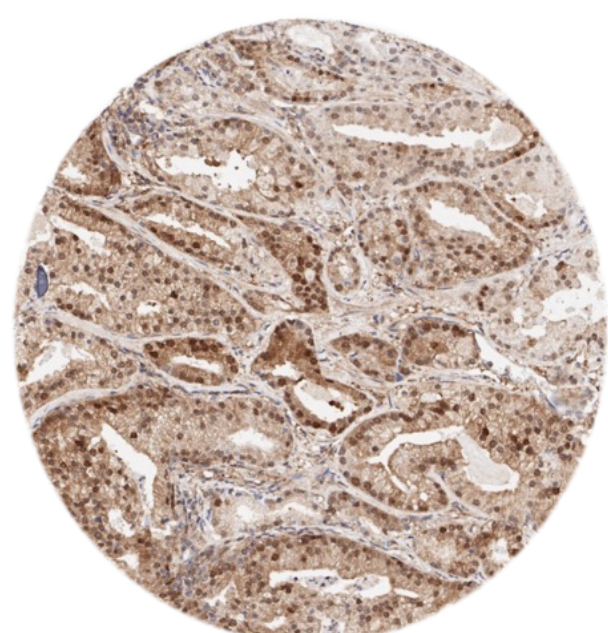

D

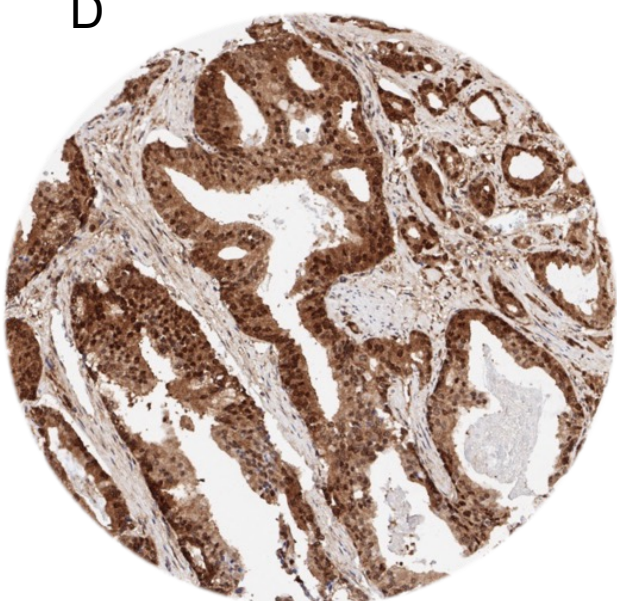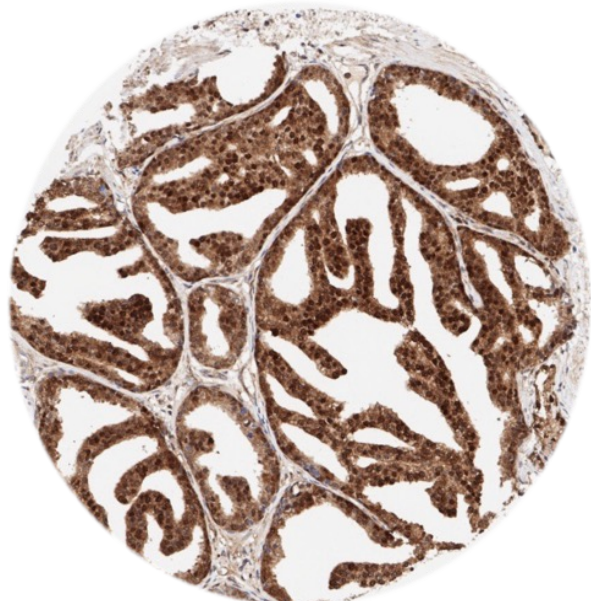

Supplement: Supplementary file 1 — Supplementary Figure 1: Examples of prostate cancers with A) negative (intensity 0), B) 1+, C) 2+ and D) 3+ staining intensity. [file mmc1.pdf]

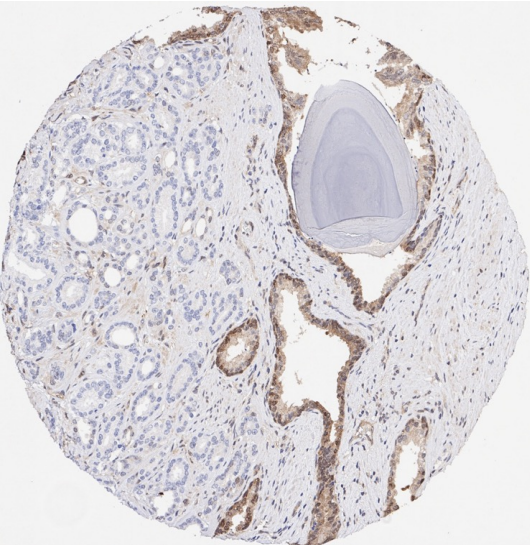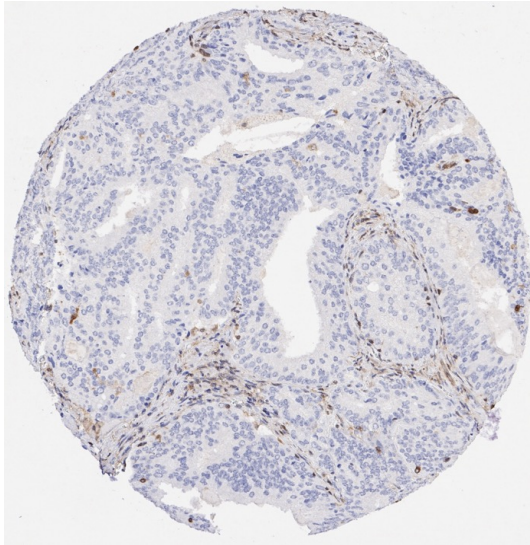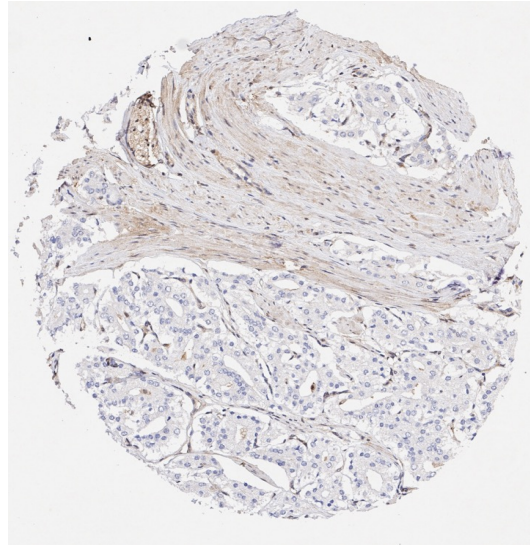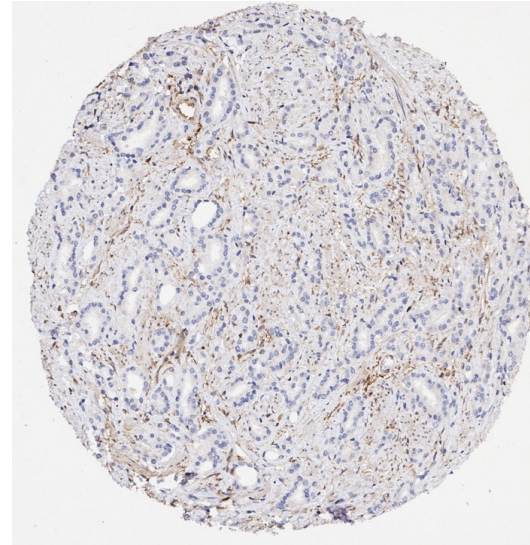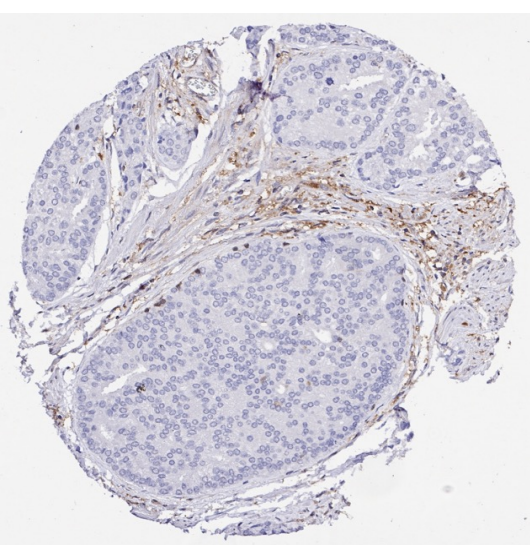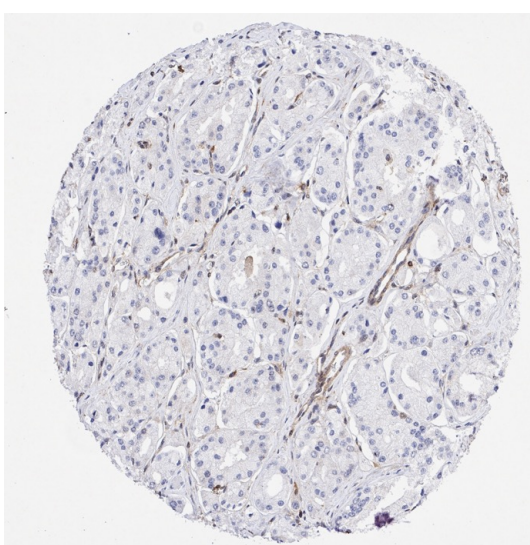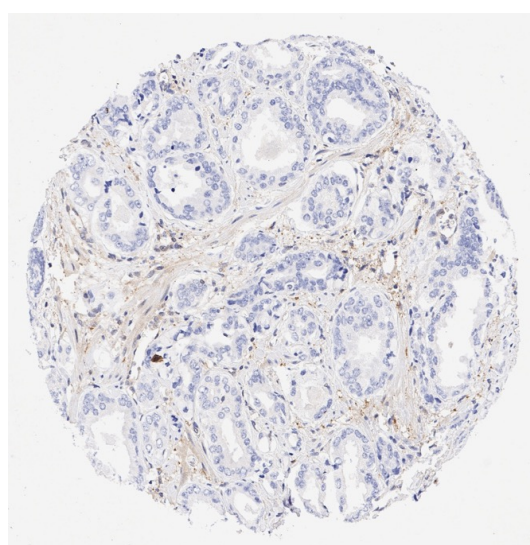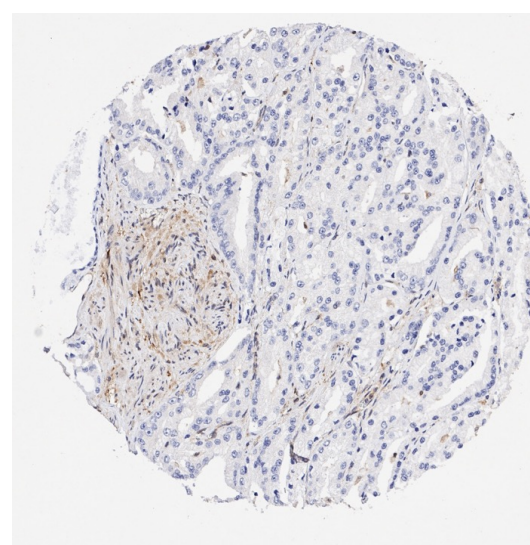

Supplement: Supplementary file 2 — Supplementary Figure 2: Further examples of prostate cancers with complete MTAP expression loss. [file mmc2.pdf]

a)

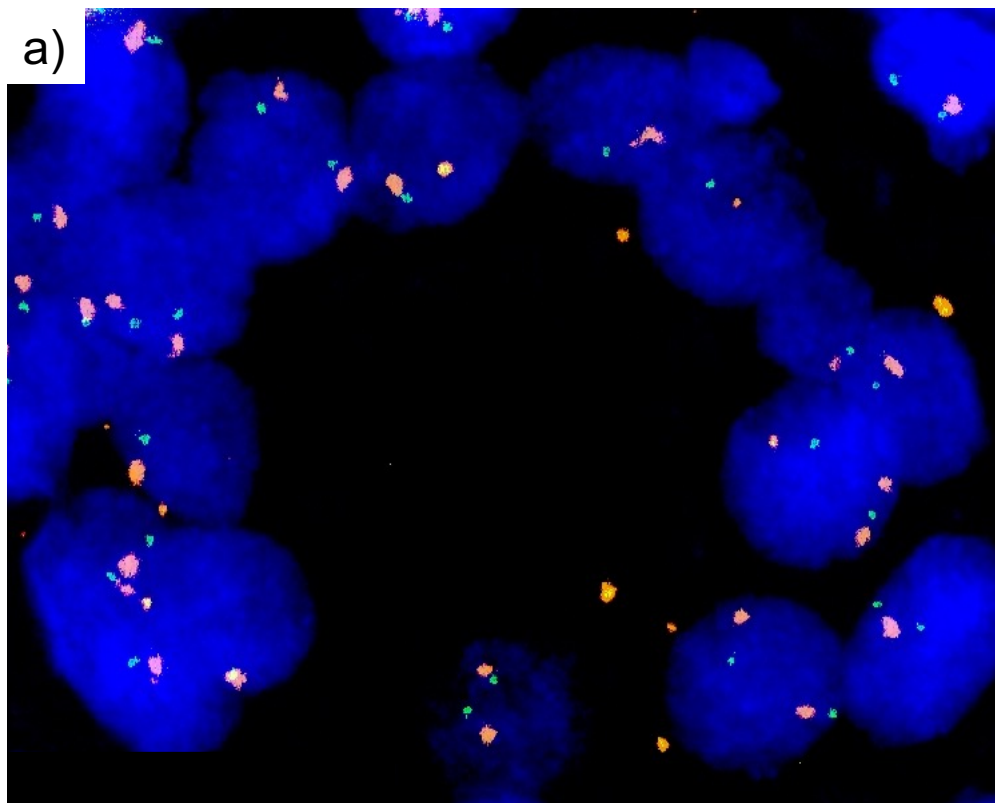

b)

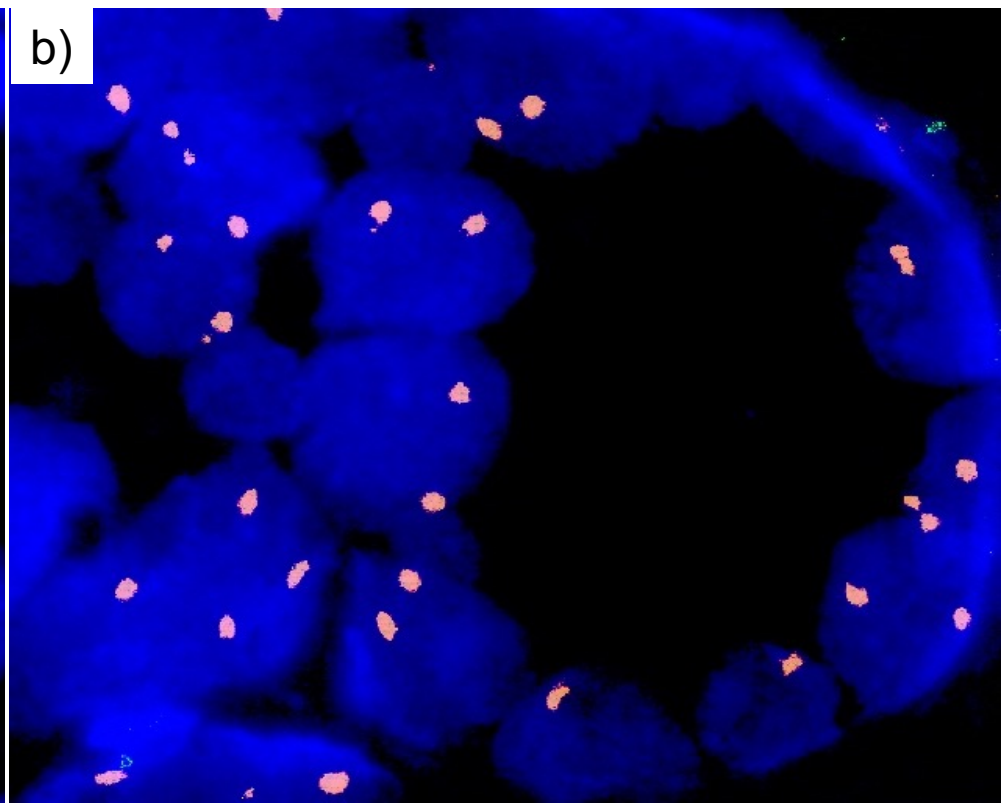

Supplement: Supplementary file 3 — Supplementary Figure 3: Examples of 9p21 FISH analysis in prostate cancer. a) normal 9p21 copy numbers as indicated by the presence of two green 9p21 locus specific probe signals and two orange centromer 9 probe signals. b) Homozygous 9p21 loss as indicated by the complete absence of the green 9p21 locus specific probe signals. Cell nuclei were couterstained with DAPI (blue color). [file mmc3.pdf]

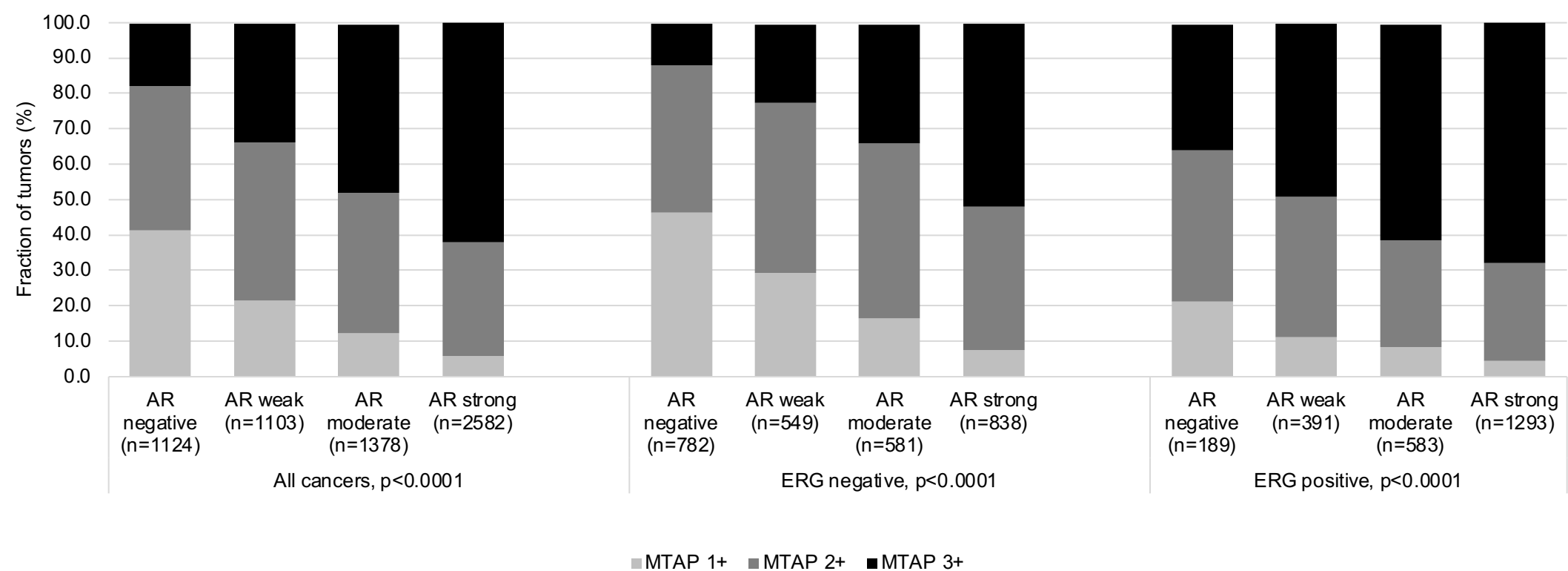

Supplement: Supplementary file 4 — Supplementary Figure 4: MTAP expression and AR expression. [file mmc4.pdf]

a)

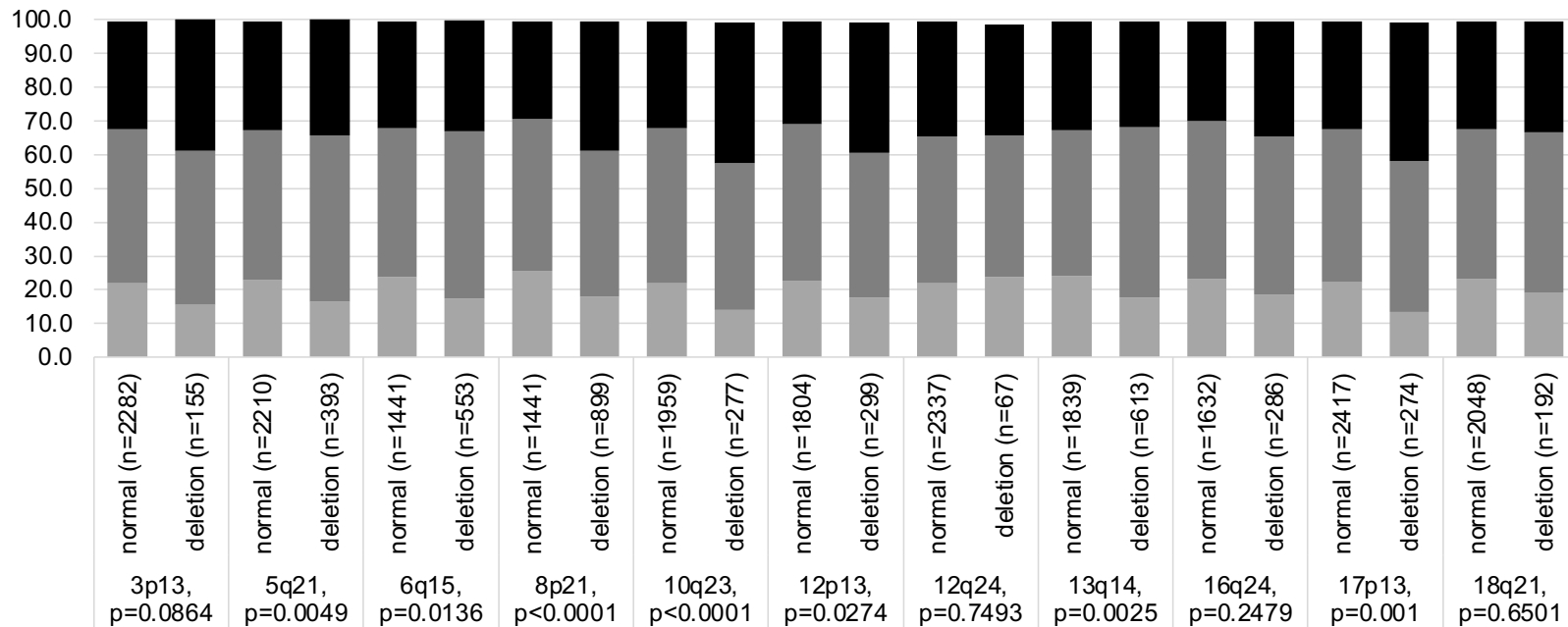

b)

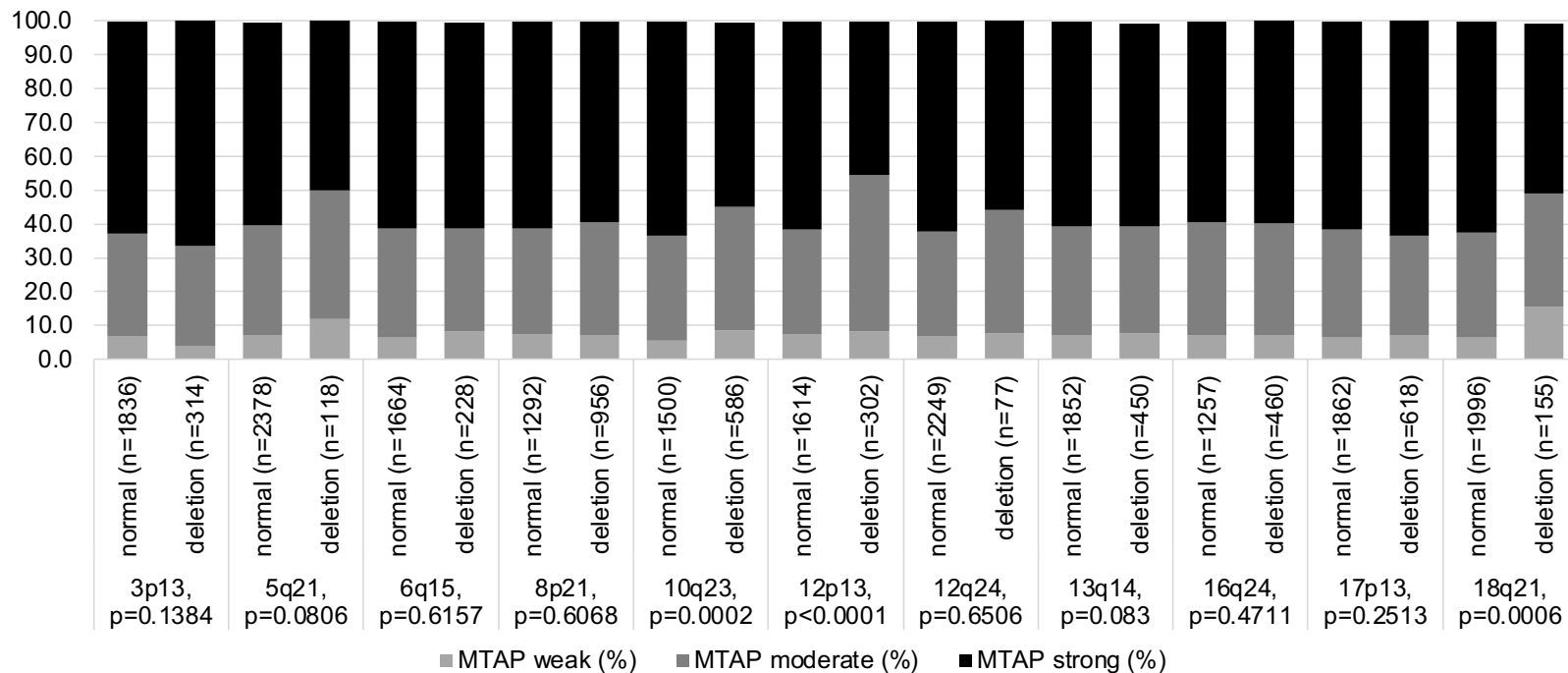

Supplement: Supplementary file 5 — Supplementary Figure 5: MTAP expression and genomic deletions in a) ERG negative and b) ERG positive cancers. [file mmc5.pdf]
