# Supplementary material for: Prognostic role of high MTAP expression is reversed by the ERG status in prostate cancer treated by radical prostatectomy
Source: Neoplasia. 2025 Jun 18;67:101197. doi: 10.1016/j.neo.2025.101197 (PMC12214127; doi:10.1016/j.neo.2025.101197)
Supplement: Supplementary file 6 — Supplementary Table 1. Study cohort [file mmc6.docx]

|  | **No. of patients (%)** | |
| --- | --- | --- |
|  | **Study cohort on TMA** | **Biochemical relapse among categories** |
|  | **(n=17,747)** |  |
| **Follow-up (mo)** |  |  |
| **n** | 14,464 (81.5%) | 3,612 (25%) |
| Mean | 56.3 | - |
| Median | 48 | - |
| **Age (y)** |  |  |
| ≤50 | 433 (2.4%) | 66 (15.2%) |
| 51-59 | 4,341 (24.5%) | 839 (19.3%) |
| 60-69 | 9,977 (56.4%) | 2,073 (20.8%) |
| ≥70 | 2,936 (16.6%) | 634 (21.6%) |
| **Pretreatment PSA (ng/ml)** | |  |
| <4 | 2,225 (12.6%) | 313 (14.1%) |
| 4-10 | 10,520 (59.6%) | 1,696 (16.1%) |
| 10-20 | 3,662 (20.8%) | 1,043 (28.5%) |
| >20 | 1,231 (7%) | 545 (44.3%) |
| **pT stage (AJCC 2002)** |  |  |
| pT2 | 11,518 (65.2%) | 1,212 (10.5%) |
| pT3a | 3,842 (21.7%) | 1,121 (29.2%) |
| pT3b | 2,233 (12.6%) | 1,213 (54.3%) |
| pT4 | 85 (0.5%) | 63 (74.1%) |
| **Gleason grade** |  |  |
| ≤3+3 | 3,570 (18.1%) | 264 (7.4%) |
| 3+4 | 9,336 (47.4%) | 1,436 (15.4%) |
| 3+4 Tert.5 | 1,697 (8.6%) | 165 (9.7%) |
| 4+3 | 2,903 (14.7%) | 683 (23.5%) |
| 4+3 Tert.5 | 1,187 (6%) | 487 (41%) |
| ≥4+4 | 999 (5.1%) | 531 (53.2%) |
| **pN stage** |  |  |
| pN0 | 10,636 (89.4%) | 2,243 (21.1%) |
| pN+ | 1,255 (10.6%) | 700 (55.8%) |
| **Surgical margin** |  |  |
| Negative | 14,297 (80.8%) | 2,307 (16.1%) |
| Positive | 3,388 (19.2%) | 1,304 (38.5%) |
| NOTE: Percent in the column "Study cohort on TMA" refers to the fraction of samples across each category. Percent in column "Biochemical relaps among categories" refers to the fraction of samples with biochemical relaps within each parameter in the different categories. Numbers do not always add up to 17,747 in the different categories because of cases with missing data. Abbreviation: AJCC, American Joint Committee on Cancer. | | |
