# Supplementary material for: Prognostic role of high MTAP expression is reversed by the ERG status in prostate cancer treated by radical prostatectomy
Source: Neoplasia. 2025 Jun 18;67:101197. doi: 10.1016/j.neo.2025.101197 (PMC12214127; doi:10.1016/j.neo.2025.101197)
Supplement: Supplementary file 7 — Supplementary Table 2: Associations between MTAP expression and prostate cancer phenotype in all cancers. [file mmc7.docx]

|  |  |  | **MTAP IHC result** | | | |  |
| --- | --- | --- | --- | --- | --- | --- | --- |
|  |  | **n evaluable** | **0+** | **1+** | **2+** | **3+** | **p value** |
| **all cancers** |  | 13189 | 0.3 | 14.8 | 42.2 | 42.7 |  |
|  |  |  |  |  |  |  |  |
| **Tumor stage** | pT2 | 8161 | 0.2 | 15.7 | 41.7 | 42.4 | 0.0004 |
|  | pT3a | 3059 | 0.4 | 12.8 | 42.1 | 44.8 |  |
|  | pT3b-4 | 1910 | 0.4 | 14.3 | 44.5 | 40.8 |  |
| **Gleason grade** | ≤3+3 | 2340 | 0.1 | 20.3 | 36.5 | 43.1 | <0.0001 |
|  | 3+4 | 6983 | 0.3 | 14.1 | 41.7 | 43.9 |  |
|  | 3+4 Tert.5 | 624 | 0.2 | 12.7 | 47.4 | 39.7 |  |
|  | 4+3 | 1355 | 0.2 | 12.8 | 45.7 | 41.3 |  |
|  | 4+3 Tert.5 | 969 | 0.2 | 11.8 | 46.1 | 41.9 |  |
|  | ≥4+4 | 799 | 0.5 | 14.3 | 46.3 | 38.9 |  |
| **quantitative Gleason** | 3+4 ≤5% | 1727 | 0.3 | 13.7 | 40.9 | 45.1 | <0.0001 |
|  | 3+4 6-10% | 1712 | 0.3 | 14.9 | 39.5 | 45.3 |  |
|  | 3+4 11-20% | 1532 | 0.3 | 12.3 | 42.5 | 44.9 |  |
|  | 3+4 21-30% | 786 | 0.1 | 12.7 | 46.3 | 40.8 |  |
|  | 3+4 31-49% | 658 | 0.3 | 17.6 | 41.3 | 40.7 |  |
|  | 4+3 50-60% | 529 | 0.2 | 12.7 | 47.4 | 39.7 |  |
|  | 4+3 61-80% | 483 | 0.2 | 11.9 | 45.6 | 42.3 |  |
|  | 4+3 >80% | 128 | 0.2 | 13.5 | 42.2 | 44.1 |  |
| **Lymph node metastasis** | N0 | 8088 | 0.2 | 14 | 43.2 | 42.6 | 0.0315 |
|  | N+ | 1035 | 0.5 | 13 | 47.2 | 39.2 |  |
| **Preop. PSA level (ng/ml)** | <4 | 1542 | 0.3 | 11.9 | 36.4 | 51.4 | <0.0001 |
|  | 4-10 | 7698 | 0.3 | 14.4 | 41.2 | 44.1 |  |
|  | 11-20 | 2830 | 0.2 | 16.5 | 45.8 | 37.5 |  |
|  | >20 | 1033 | 0.1 | 18 | 47.9 | 34 |  |
| **Surgical margin** | negative | 10380 | 0.3 | 15 | 41.7 | 43.1 | 0.2455 |
|  | positive | 2755 | 0.3 | 14.3 | 43.9 | 41.6 |  |
