# Supplementary material for: Prognostic role of high MTAP expression is reversed by the ERG status in prostate cancer treated by radical prostatectomy
Source: Neoplasia. 2025 Jun 18;67:101197. doi: 10.1016/j.neo.2025.101197 (PMC12214127; doi:10.1016/j.neo.2025.101197)
Supplement: Supplementary file 8 — Supplementary Table 3: Multivariate analysis. Cox proportional hazards regression p-values are shown for established prognostic parameters and MTAP expression in different clinical scenarios and in subsets of ERG-negative and ERG-positive cancers. [file mmc8.docx]

| **Tumor subset** | **Scenario** | **n analyzable** | **p -value** | | | | | | | |  |
| --- | --- | --- | --- | --- | --- | --- | --- | --- | --- | --- | --- |
|  |  |  | **preoperative PSA-Level** | **pT Stage** | **cT Stage** | **Gleason grade prostatectomy** | **Gleason grade biopsy** | **pN stage** | **R status** | **MTAP-Expression** |  |
|  |  |  |  |  |  |  |  |  |  |  |  |
| **ERG negative cancers** | 1 | 3,039 | <0.0001* | <0.0001* | - | <0.0001* | - | <0.0001* | 0.1992 | 0.0115 |  |
|  |  |  |  |  |  |  |  |  |  |  |  |
|  | 2 | 4,511 | <0.0001* | <0.0001* | - | <0.0001* | - | - | 0.0022* | 0.0035* |  |
|  |  |  |  |  |  |  |  |  |  |  |  |
|  | 3 | 4,447 | <0.0001* | - | <0.0001* | <0.0001* | - | - | - | 0.0001* |  |
|  |  |  |  |  |  |  |  |  |  |  |  |
|  | 4 | 3,742 | <0.0001* | - | <0.0001* | - | <0.0001* | - | - | 0.0003* |  |
| **ERG postive cancers** | 1 | 2,471 | <0.0001* | <0.0001* | - | <0.0001* | - | 0.0030* | <0.0001* | 0.0922 |  |
|  |  |  |  |  |  |  |  |  |  |  |  |
|  | 2 | 3,765 | <0.0001* | <0.0001* | - | <0.0001* | - | - | <0.0001* | 0.1907 |  |
|  |  |  |  |  |  |  |  |  |  |  |  |
|  | 3 | 3,693 | <0.0001* | - | <0.0001* | <0.0001* | - | - | - | 0.0708 |  |
|  |  |  |  |  |  |  |  |  |  |  |  |
|  | 4 | 3,189 | <0.0001* | - | <0.0001* | - | <0.0001* | - | - | 0.0401 |  |
